# Characterization of noise in long-term ECG monitoring with machine learning based on clinical criteria

**DOI:** 10.1007/s11517-023-02802-5

**Published:** 2023-04-03

**Authors:** Roberto Holgado-Cuadrado, Carmen Plaza-Seco, Lisandro Lovisolo, Manuel Blanco-Velasco

**Affiliations:** 1 Department for Signal Theory and Communications, Universidad de Alcalá, 28800 Alcalá de Henares, Madrid Spain; 2 DETEL - Dep. of Electronics and Communications Engineering, UERJ - Rio de Janeiro State University, Rio de Janeiro, Brazil

**Keywords:** Clinical noise severity, Electrocardiogram (ECG), Long-term monitoring (LTM), Machine learning (ML), Signal quality

## Abstract

Noise and artifacts affect strongly the quality of the electrocardiogram (ECG) in long-term ECG monitoring (LTM), making some of its parts impractical for diagnosis. The clinical severity of noise defines a qualitative quality score according to the manner clinicians make the interpretation of the ECG, in contrast to assess noise from a quantitative standpoint. So clinical noise refers to a scale of different levels of qualitative severity of noise which aims at elucidating which ECG fragments are valid to achieve diagnosis from a clinical point of view, unlike the traditional approach, which assesses noise in terms of quantitative severity. This work proposes the use of machine learning (ML) techniques to categorize different qualitative noise severity using a database annotated according to a clinical noise taxonomy as gold standard. A comparative study is carried out using five representative ML methods, namely, *K* neareast neighbors, decision trees, support vector machine, single-layer perceptron, and random forest. The models are fed by signal quality indexes characterizing the waveform in time and frequency domains, as well as from a statistical viewpoint, to distinguish between clinically valid ECG segments from invalid ones. A solid methodology to prevent overfitting to both the dataset and the patient is developed, taking into account balance of classes, patient separation, and patient rotation in the test set. All the proposed learning systems have demonstrated good classification performance, attaining a recall, precision, and F1 score up to 0.78, 0.80, and 0.77, respectively, in the test set by a single-layer perceptron approach. These systems provide a classification solution for assessing the clinical quality of the ECG taken from LTM recordings.

**Graphical Abstract:**
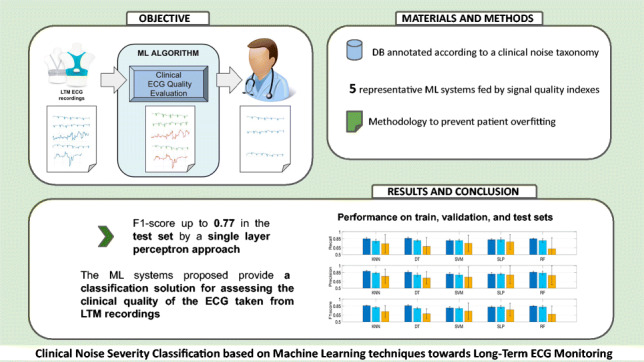
Clinical Noise Severity Classification based on Machine Learning techniques towards Long-Term ECG Monitoring

Clinical Noise Severity Classification based on Machine Learning techniques towards Long-Term ECG Monitoring

## Introduction

The electrocardiogram (ECG) has proven to be reliable and effective for studying the electrical activity of the heart [1]. Standard 12–leads ECG is collected for seconds or minutes by attaching electrodes to the patient’s skin [2], while 24–48-h Holter ECG provides longer recordings to track events that are not found in short-term ECG, such as several types of arrhythmias [3, 4]. Under the suspicion of intermittent pathologies, manifested as occasional transients, monitoring the heart activity for extended periods of several days is becoming apparent [5]. Such ECGs are called long- term monitoring (LTM) recordings, they are acquired for 7, 15, or even 21 days during daily routine activity, and they have been found to be very useful to detect atrial fibrillation in patients with cryptogenic stroke [6] and atrial or ventricular subclinical arrhythmias [7]. Due to the ambulatory registering of LTM recordings and their long duration, some parts of the ECG are severely contaminated by white or colored noise, narrow-band interference (from powerline and other sources impinging the sensor) [8], patient movements, baseline oscillation, electromyographic electrical activity, electrode movement, and many other equipment problems, thus resulting close to impractical for diagnosis. So the identification of useless segments from a clinical standpoint would be beneficial not only to improve the performance of signal processing systems, but also to reduce the time required to analyze recordings by human operators. Notice that traditional ECG signal processing methods and clinical analysis are not adapted to deal with the huge amount of data gathered by LTMs, demanding the design of appropriate protocols and techniques to provide robust results.

From an engineering point of view, the impact of noise in the ECG is assessed in terms of a figure of merit, e.g., the signal-to-noise ratio (SNR), which indicates how severe is the noise which contaminates the ECG. Nonetheless, the quantitative severity of noise not necessarily informs about the validity of an ECG to provide a diagnosis. This is because the interest of cardiologists relies on the readability of the P, Q, R, S, T subwaves of a heartbeat. Thus, it may occur that an ECG beat with high SNR, i.e., with small quantitative noise severity, lacks some subwaves, been considered in such case as non-valid or very limited from a clinical viewpoint, despite the small quantitative severity informs that the impact of noise is minimal. So, this is an example where an ECG excerpt with very low quantitative noise severity is, conversely, defined as with high severity from a clinical standpoint. In other words, one of the main drawback of the quantitative approach is that current metrics have no clinical meaning, so they are not useful for physicians because they cannot interpret them. On the other hand, modeling noise from a clinical viewpoint is an extremely difficult issue, which has not yet been fruitfully addressed. However, if the cardiologists’ knowledge is successfully transferred to a database, any machine learning (ML) method will be able to mimic the clinical expert behavior. So, the problem of noise in this work is faced by adopting the clinical point of view.

The clinical severity of noise is defined as the impact that noise causes in the clinical interpretation of ECG segments to establish a diagnosis. This severity is not assessed by quantitative means, but by a set of rules that a cardiologist follows to make the interpretation of the ECG. We refer noise in this context as clinical noise. An ECG fragment is said to be of good clinical quality (noiseless from clinical noise viewpoint) when the clinical patterns to make a diagnosis are visible in the wave shape. Thus, clinical noise severity is assessed in a qualitative scale. In [9, 10], a 5-level scale scoring the effect of clinical noise on the ECG was designed based on the recommendations given by a team of cardiologists. A methodology, based on noise maps, to evaluate how well qualitative severity correlates with quantitative severity was also developed. A manually annotated database of actual LTM recordings according to the scale was used as gold standard. The work concluded that qualitative and quantitative severity are not correlated, showing that quantitative noise indices may not be appropriate to determine clinical quality in electrocardiography and LTM recordings.

Regarding earlier works, many of them drive their attention to ECG enhancement [11–14], though they rely on quantitative metrics, and the result may not be necessarily useful for clinical purposes due to ECG pattern degradation [10]. Another alternative approach is estimating the quality of the ECG employing quantitative quality indexes, such as the rate of change of the spectral components [15] or the number of R–peaks [16]. Several works use a binary scale assessed by means of signal processing techniques to detect possible disturbances [17], and time-frequency analysis [18], or heuristic-based approaches [19]. Others rest on ML algorithms fed with features derived from the signal autocorrelation [20, 21], the eigenvalues of the covariance matrix between leads [22], or signal quality indexes derived from statistics [23], and ECG morphological aspects [24, 25]. Meanwhile, multi-level quality scales are found as well [26, 27]. For most methods, the validation methodology relies on the annotation of short-term ECG segments during few seconds and the addition of noise of different levels for varying the SNR.

Regarding databases, most of the contributions deal with datasets consisting of isolated ECG segments with artificially added noise as an intention to replicate real working conditions. Realistic recordings taken from public databases are used in combination with synthetically generated noise such as in [28, 29]. In [24, 26] authors bootstrap the unrepresented class by adding noise to the clean data to balance the dataset, while [15] uses also ECGs containing real noise. Synthetic noise is employed in [25] in a single-patient noisy database, and similarly, synthetic and real noise ECG are used in [27]. In clinical noise, the qualitative assessment of its impact impairs the development of synthetic databases.

The present work seeks to find out whether clean and noisy ECG segments defined in a qualitative scale can be distinguished by ML. We rely on the fact that learning-based methods have proven to perform very well in tasks that are easily executed by human operators, but that are very difficult to model mathematically. To achieve this, we rely on cardiologists’s experience, which has been transferred to a database according to the set rules defining a taxonomy of clinical noise. Thus, distinction between classes defined on a qualitative severity scale is the main novelty of this research, and the contribution is the development of systems to provide a classification solution for assessing clinical quality of ECGs taken from LTM recordings. An ML-based methodology is carried out, where a set of features describing the waveform and the information content of the ECG are used as predictors. A comparison study of several systems is addressed into a continuously running labelled database defined to be our gold standard for clinical noise. The results obtained show that the identification of clinically clean signal frames is possible, and that these ML methods can learn from this data to attribute a scale of clinical noise severity.

This paper is organized as follows. Section 2 introduces the database, its segmentation, and the ML methods applied to classification. Feature engineering and the experimental setting are reported in Section 3. The results are presented in Section 4, and the discussion and conclusions of this work are finally addressed in Sections 5 and 6, respectively.

## Materials and methods

### Data gathering and annotation

It has been shown in previous studies that there exist apparent differences on how the impact of noise affects to the interpretation of the ECG when it is assessed by quantitative means than when the interpretation is made by cardiologist [9, 10], i.e., qualitatively. The former approach relies on quantitative noise severity, based on figures of merit. The latter one relies on the knowledge of cardiologist about the requirement of the ECG waveform to contain sufficient information for a complete interpretation. Any factor preventing the right ECG interpretation can be considered as noise, and because the latter perspective is only based on cardiologists’ knowledge, we refer it to as clinical noise. The measure of the impact of clinical noise into the ECG interpretation is qualitative, which we refer to as clinical noise severity. In the current work we deal with the interpretation of the ECG as understood by cardiologist, so with clinical noise. In [9, 10], a noise taxonomy in terms of clinical noise was proposed in a scale of 5 categories as follows:

- *Noise-free* or type 0 (T0): segment without noise.
- *Low noise* or type 1 (T1): some noise is present in the segment, but P and T waves (corresponding to atrial depolarization and ventricular repolarization, respectively) and the QRS complexes are readable and their morphology can be identified.
- *Moderate noise* or type 2 (T2): a noisy segment in which only the QRS complexes are reliably identified, in at least three consecutive beats.
- *Hard noise* or type 3 (T3): noisy segment with hardly recognizable or unrecognizable QRS complexes.
- *Other noise* or type 4 (T4): segments are calibration pulses or straight lines because of the complete absence of signal or amplifier saturation.

This set of rules expresses the characteristics that an ECG must carry to be valid for a clinical analysis. After jointly analyzing this 5-level scale from a clinical view, we infer that at least the QRS complex plus another additional subwave (P or T) must be present to be considered as type 0 or type 1. The difference is that although the same pattern must be identifiable, type 1 tolerates any sort of artifacts, no matter the size, as long as the heartbeat morphology can be read. Conversely, types 2 and 3 are categories describing ECGs with limited diagnostic capacities. This can be caused by numbers of reasons: absence of QRS complex, absence of both P and T waves, or because there is no trace of readable ECG in the signal. Cardiac rhythm can be identified in type 2, because QRS complex continuity is required in at least 3 beats, but type 3 represents a worthless signal from a clinical viewpoint. Beyond these four categories, there is an additional one representing non signal presence.

**Fig. 1:**
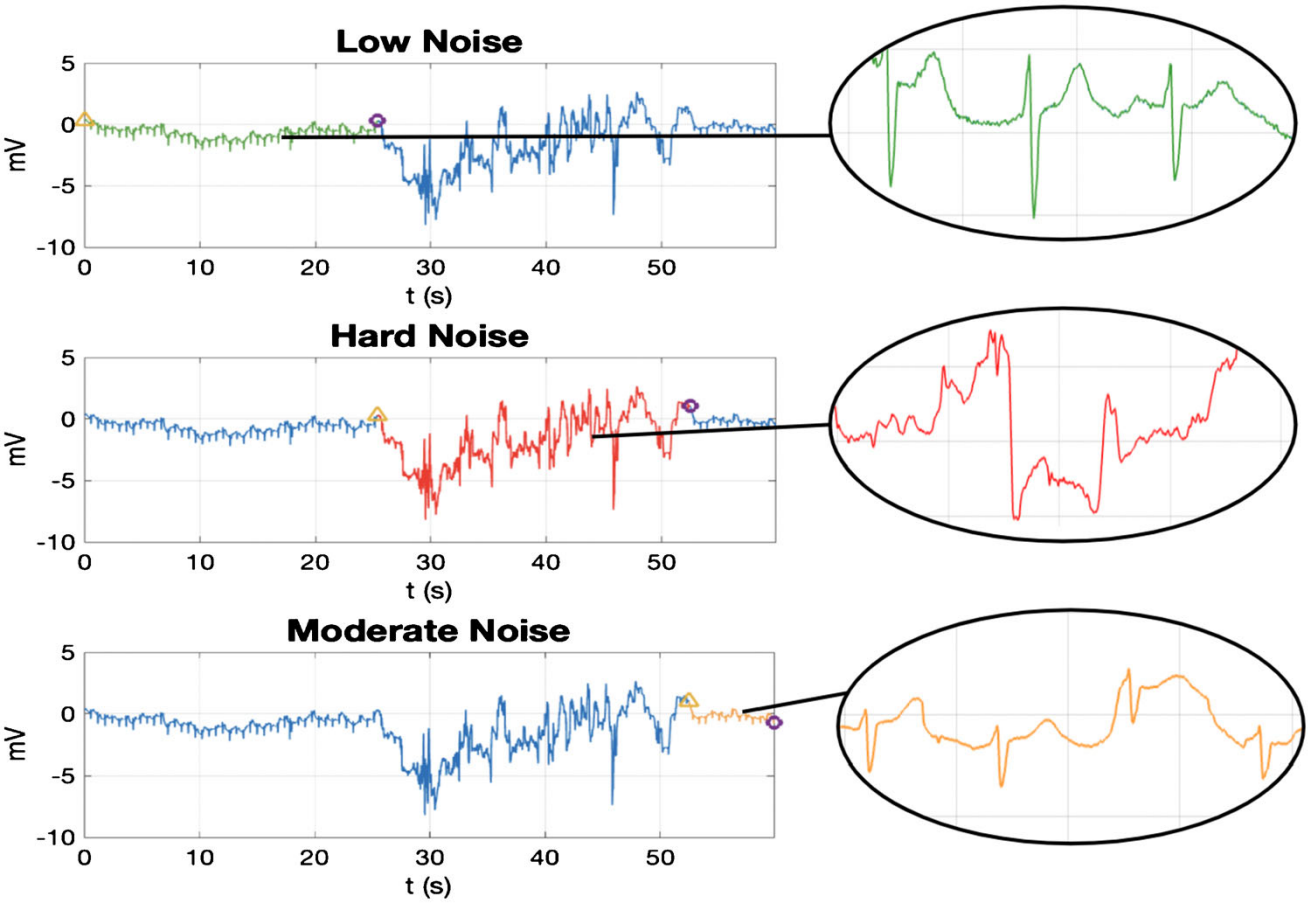
Labelling recording example from the original clinical noise database taken from an EER. The signal goes through 3 categories highlighted in each graph (from top to bottom): *low noise* (T1), *hard noise* (T3), and *moderate noise* (T2). The yellow triangle indicates the segment onset and the purple circle the offset

According to these criteria, a set of data acquired (*f*_*s*_ = 200 Hz) with an External Event Recorded (EER) was continuously labeled throughout its entire duration. More than 6.5 h of recording from 10 patients distributed in 250 recordings of about 30 to 300 s, 2 leads per recording were annotated (see [9, 10] for more details). Figure 1 illustrates one of these recordings as an example of the the manner the signals are labeled and the how the ECG signals in each category look like. The ECG excerpt lasts 60 s approximately, and contains three clinical noise categories: transition between classes T1 to T3 is around *t* = 25 s, and from T3 to T2 is around *t* = 52 s. A closer look at the waveform exhibits how the hard noise class (T3), in the middle panel, has lost the regular ECG beat pattern, and can scarcely be used to extract clinical information. Since clinical noise relies on qualitative noise assessment, we only account on this material to define the experimental framework, where training systems on real ECG, manually annotated, is among the main challenges.

### Materials

Although clinical noise is split in several groups, the proposed learning approach is faced as a binary problem to distinguish from only 2 classes: *clean* and *noisy*. We consider as *noisy* the classes which convey less clinical information: for this work we have chosen *moderate noise* (T2) and *hard noise* (T3). Thus, *noise–free* (T0) and *low noise* (T1) are enclosed into the *clean* class. We have chosen to include T2 in the *noisy* class since the lack of P and T waves prevents important information related to atrial depolarization and ventricular repolarization. Finally, T4 (*other noise*) has not been taken into account because this is a category which explains recording excerpts with no signal, which can easily be identified by simple signal processing techniques (see Table 1 for class distribution).

**Table 1:** Database size and distribution per classes (*clean* or *noisy*) and noise type (*noise-free* as T0, *low noise* as T1, *moderate noise* as T2 and *hard noise* as T3) for 5-s length ECG excerpts

Class	*#* Instances	Noise type	*#* Instances
Clean	3.867 (57.31%)	T0	2.236 (33.14%)
		T1	1.631 (24.17%)
Noisy	2.881 (42.69%)	T2	2.204 (32.66%)
		T3	677 (10.03%)

To obtain instances of the same length to train and test learning models, the entire EER database is segmented, resulting in signal blocks of the same duration *s*(*t*), 0 ≤ *t* ≤ *t*_*b*_, and of one single category. They can be regarded as a bunch of *L* discrete samples enclosed into the vector

1 $$ \textbf{s}^{(i)}= \left[ s_{0}^{(i)},s_{1}^{(i)}\cdots,s_{L-1}^{(i)}\right]^{T},~i = 1,\cdots,m $$

where *i* stands for the *i* th example or instance, and *m* is the size of the dataset. To ensure consistent and correct methodology, the following conditions have been hold in the segmentation process: (a) independence among segments **s**^(*i*)^ by choosing non-overlapped blocks; (b) maximal difference among instances by leaving a time gap between consecutive blocks.

The block length *t*_*b*_ is a critical parameter since all the classes must be represented into each instance. Although the categories are defined in a heartbeat dimension, *moderate noise* (T2) definition accounts for at least 3 consecutive beats. So, a block length from 3 up to 6 s is reasonable to ensure 3 beats in a segment. In this work, we have taken *t*_*b*_ = 5 s.

**Fig. 2:**
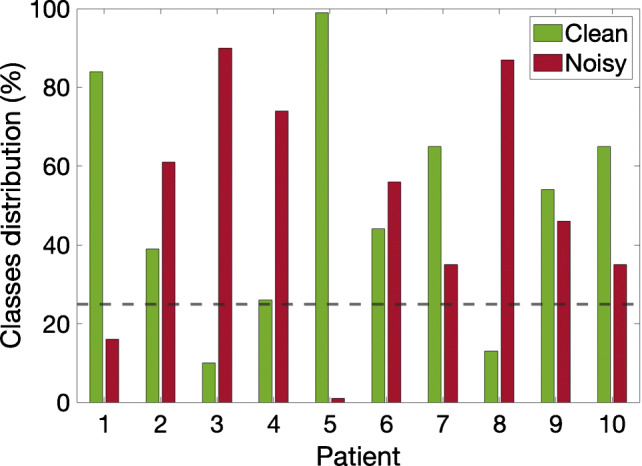
Distribution of *clean* and *noisy* classes per patient. Patients with any class below 25% are discarded for the train and validation process

A total of 6748 examples as shown in Table 1 are obtained, with reasonable balance between *clean* and *noisy* classes. However, there is strong imbalance when regarded by patients, as can be seen in Fig. 2. Patients 1, 3, 5, and 8 may be unsuitable because the models can have difficulty to learn from them. For this reason, we have decided to discard patients presenting less than 25% of instances in any class, which reduces the set to only 6 patients for the classifier design, namely, patients 2, 4, 6, 7, 9, and 10. Operating in this manner reduces the set of instances to *m* = 2818 (47% *clean* and 53% *noisy*).

### Methods

In this study, ML methods are used to identify valid ECG frames to extract useful clinical information. Support vector machines (SVM), multi-layer perceptron (MLP) and decision tree (DT) have been used in noise classification [22, 24, 26]. So, we are going to implement these models also in this work. In addition and for comparison purposes, *K*-nearest neighbors (*K* NN) and random forest (RF) are also considered. This choice is a good representation of ML models to perform an experiment on a lower to a higher scale of flexibility. Hyperparameter tuning is performed with bayesian optimization, except for neural networks, which employ grid search [30, 31]. This section introduces a brief review of these methods.

The first algorithm, which is based on a similarity measure, is *K* NN. *K* is the number of neighbors. A test observation **x** is classified by the vote of its neighbors (*K* in the training set that are closest to **x**), being assigned with the most common class among all its neighbors [32, 33]. With the Euclidean metric as distance metric, the hyperparameter that is optimized in this work is *K*.

A second approach involves the DT, which is an algorithm that assigns the output variable using the decision rules learned from training datapoints [33–35]. To control the depth of the tree and to avoid overfitting to the DT, the hyperparameter to be optimized is the minimum number of samples at each leaf node.

Moving on to more complex models, we find SVM, which is an algorithm whose classification strategy is based on optimally separating a set of data by a hyperplane or set of hyperplanes. In this case, linear SVM is employed and the hyperparameters to be optimized are a scaling parameter and a regularization parameter.

With respect to artificial neural network (ANN), a simple architecture is applied, the single-layer perceptron (SLP). It is the most straightforward feedforward neural network architecture employing only one hidden layer of neurons [32, 33]. Gradient descent backpropagation is the training technique and L2-regularization [36] is applied to avoid overfitting. The hyperparameter that is optimized is the number of neurons in the hidden layer.

Finally, the RF algorithm is an algorithm that employs an ensemble of different decision trees. The trees are built independently using a small subset of the dataset randomly drawn. The features for the trees are selected randomly as well. Each tree in the RF makes a classification and the final decision is made by majority vote [33, 34]. The hyperparameters to be optimized are the minimum number of samples at each leaf node, the number of features employed in the tree branches and the number of trees in the ensemble.

## Experimental framework

### Feature engineering

After database segmentation described in Section 2, the dataset consists of a bunch of ECG blocks referred to as **s**^(*i*)^, all of the same length (5-s long), as described in Eq. 1, where *i* = 1,⋯ ,*m*, stands for the *i* th example (*m* = 2818). All blocks are preprocessed as follows. Baseline wander removal is accomplished using cubic splines interpolation: A non-overlapped sliding window of 1.2-s length is used to determine the knots (median of the window) from which the drift component is estimated; baseline wander is cancelled by subtracting the estimated drift. Powerline interference reduction is addressed by linear notch filtering (50 Hz). Bandpass filtering is applied from 0.5 up to 40 Hz to preserve the ECG spectral band (cascading two 5th order Butterworth highpass and lowpass filters). Finally, each block **s**^(*i*)^ is normalized:

2 $$ \textbf{s}^{(i)}:=\frac{\textbf{s}^{(i)}-\mu_{s}^{(i)}}{\sigma_{s}^{(i)}} $$

where symbol “:=” stands for assignment; and $\mu _{s}^{(i)}$ and $\sigma _{s}^{(i)}$ stand for the mean and standard deviation of the *i* th signal block, respectively.

This work is faced as a multidimensional classification problem where the hypothesis function is fed by multiple variables **x** = [*x*_1_,*x*_2_,⋯ ,*x*_*n*_]^*T*^ $\in \mathbb {R}^{n}$. Sixteen features (*n* = 16) were selected to represent the structure of each signal block. The variables *x*_*j*_ are listed on Table 2 and they correspond to features informing about: critical points location and distribution (*j* = 1,2); time domain characteristics (*j* = 3,4); statistical properties (*j* = 5,⋯ ,11); and spectral characteristics ($j=12,\dots , 16$). Some of them were inspired by [26]. Others, such as *x*_5_ up to *x*_8_, and *x*_11_, are well known statistics. The Spectral Purity Index (*x*_10_) is a measure of how well a signal is approximated by a pure frequency; details can be found in [37]. Feature *x*_2_ characterizes the periodic pattern of the ECG segment *s*(*t*), providing a measure of periodicity assessed as the correlation coefficient between *s*(*t*) and a shifted rotated $\tilde {s}(t) = s\left \lbrace \left (\left (t-{{\varDelta }} t_{rr}\right )\right )_{L}\right \rbrace $ of itself, where $\left (\left (\cdot \right )\right )_{L}$ stands for modulo operation. The time lag *Δt*_*rr*_ corresponds to an *RR* time interval value. So, variable *x*_2_ is obtained as:

3 $$ \rho= \frac{\textbf{s}^{T} \cdot{\tilde{\mathbf{s}}}}{||\textbf{s}||\cdot||{\tilde{\mathbf{s}}}||} $$

where $\tilde {\mathbf {s}}$ is the vector notation for $\tilde {s}(t)$. Feature *x*_15_ is the central frequency or spectral centroid:

4 $$ \overline{f}_{s}=\frac{{\int}_{0}^{\frac{f_{s}}{2}}{f P_{ss}(f)}df}{{\int}_{0}^{\frac{f_{s}}{2}}{ P_{ss}(f)df}}, $$

where *P*_*ss*_(*f*) is the power spectral density of *s*(*t*). Finally, feature *x*_16_ is the *MS-QI*. The *MS-QI* was conceived as a means to assess the changes of the ECG components, assuming that the changes rates differ between clean and noisy segments (see [15] for details).

**Table 2:** Feature list and description

Feature	Notation elsewhere	Description
*x*_1_	$A_{cc}^{R}$	R-wave Detection Accuracy [26]
*x*_2_	*ρ*	Correlation-based Periodicity
*x*_3_	$\overline {s}_{bw}$	BW check [26]
*x*_4_	$\overline {s}_{hf}$	Relative amplitude of HF noise [26]
*x*_5_	*μ*_*s*_	Mean
*x*_6_	*σ*_*s*_	Standard Deviation (STD)
*x*_7_	*β*_1_	Skewness
*x*_8_	*β*_2_	Kurtosis
*x*_9_	$\overline {\sigma }_{qrs}$	Relative STD of QRS [26]
*x*_10_	*SPI*	Spectral Purity Index [37]
*x*_11_	*H*(*s*)	Sample Entropy
*x*_12_	$\overline {p}_{bw}(s)$	Relative Power of the BW [26]
*x*_13_	$\overline {p}_{qrs}(s)$	Relative Power of the QRS [26]
*x*_14_	$\overline {e}_{qrs}(s)$	Relative energy of QRS [26]
*x*_15_	$\overline {f}_{c}(s)$	Central Frequency
*x*_16_	*MS–QI*	ECG Quality Index [15]

**Fig. 3:**
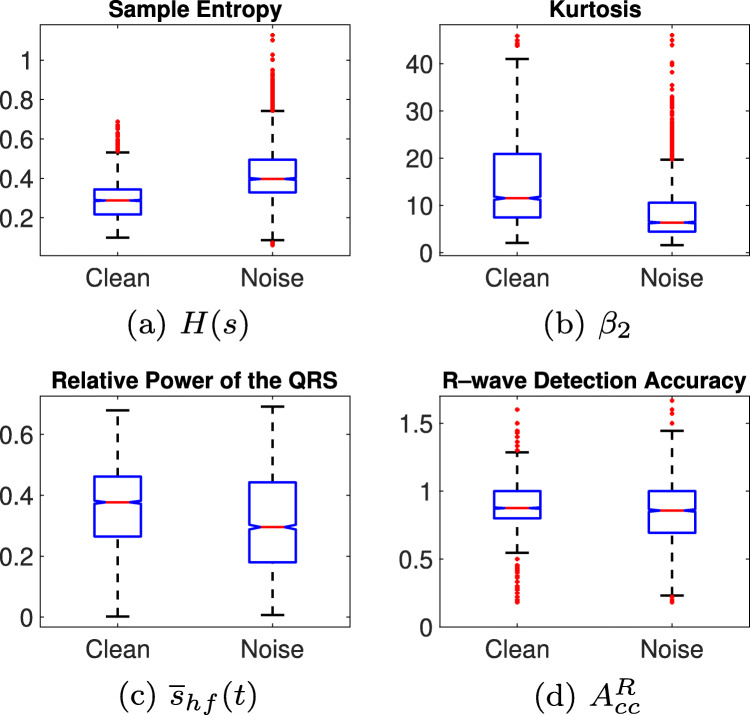
Examples of features distribution. The features in (a) and (b) exhibit some inter-class separation, while the ones in (c) and (d) are severely overlapped

A close analysis of the feature distributions over the classes reveals severe inter-class overlap, which, on the one hand, makes impossible straight separation, and, on the other hand, can even impair classification from learning systems. As representative examples of that, Fig. 3a and b show the boxplots for the two features which better separate classes, whereas Fig. 3c and d are two examples of strong overlap. So, to shed some light on the feasibility of these features for class separation, hypothesis testing is used to analyze differences between the series coming from the two classes [38]. First, a *χ*^2^-Goodness-of-Fit test is used to determine whether variables are likely to come from a normal distribution, so in those variables resulting significant to the test (all except *x*_3_ and *x*_5_), the alternative hypothesis that their distribution is non–normal is accepted and a Wilcoxon–Mann–Whitney (WMW) *U*-Test test is applied to see whether they have different median. All variables are significant to the WMW test (*α* = 0.05 and *p* < 0.05), except *SPI* (*p* = 0.31). Alternatively, a *t*–test is applied to *x*_3_ and *x*_5_, where only the former results significant, accepting thus the alternative hypothesis that classes have different mean. So, from the hypothesis testing, we can conclude that despite huge inter-class overlap, features are distributed differently within each class in the majority of cases.

In summary, features $\textbf {x}^{(i)} =\left [x_{1}^{(i)},x_{2}^{(i)}, \cdots , x_{n}^{(i)}\right ]^{T}$ are extracted from each ECG block **s**^(*i*)^, arranging a dataset for the classification task consisting of a matrix $\textbf {X}\in \mathbb {R}^{m\times n}$, where *m* = 2818 and *n* = 16. Each entry of the matrix (row-wise) corresponds to one example associated to one of the two clinical noise categories (*clean* or *noisy*) in the target vector $\textbf {y}=\left [y^{(1)},y^{(2)},{\cdots } y^{(m)}\right ]^{T}$. So, the *i* th instance is given by the pair: $\left (\textbf {x}^{(i)},y^{(i)} \right )$. Feature scaling is carried out through *z*-score normalization:

5 $$ z_{j}^{(i)}= \frac{x_{j}^{(i)}-\mu_{j}}{\sigma_{j}},~j = 1,\cdots,n $$

where *μ*_*j*_ and *σ*_*j*_ stand for the mean and the standard deviation of feature *x*_*j*_ from the train set.

### Experimental setting

Any trained model learns the ECG characteristics of patients inside the train set, so if they are are shared in the test set, the performance model can be overestimated. This phenomenon, referred to as intra–patient overfitting hereafter, will be prevented by never allocating instances from patients of the train set in the test set.

**Fig. 4:**
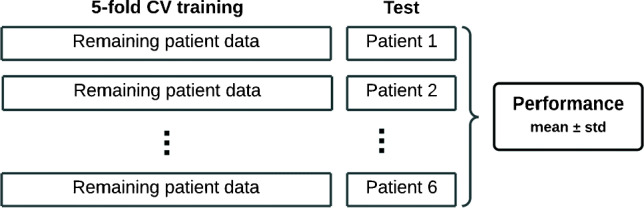
Validation scheme, where all the instances of one single patient are included in test set. The remaining instances are used to perform 5-fold CV and hyperparameter tuning. The split is random, 80% and 20 % to train and validation, respectively. In total, six models are tested

To compensate the low availability of patients, 6 different models are designed by letting one patient out in the test set to estimate their performance, as shown in Fig. 4. This procedure also minimizes intra-patient overfitting. The patients not allocated in the test set are used to design the model by performing 5-fold cross-validation (CV) on 80% of the randomized instances in the training set, and the remaining 20% in the validation set for hyperparameter tuning. Thus, one optimal model per each patient in the test set is designed, and their hyperparameter values are shown in Table 3. The overall estimated performance is calculated as the mean of all combinations, and the standard deviation is used as an indicator of consistency.

**Table 3:** Hyperparameters value of the optimal ML models

	KNN	DT	SVM		SLP	RF		
Patient test	*K*	*mSL*	*S*	*C*	*neurons*	*mSL*	*nFeats*	*nTrees*
**2**	11	11	21.92	990.51	55	51	5	100
**4**	15	77	16.06	994.56	40	50	13	150
**6**	11	23	3.34	39.83	50	50	12	50
**7**	13	79	0.14	0.13	55	50	15	250
**9**	11	22	0.81	3.73	60	50	15	100
**10**	37	20	1.80	12.26	55	50	13	150

*K* is the number of neighbors, *mSL* is the minimum samples at each leaf node, *S* is a scaling factor, *C* is the regularization parameter, *neurons* is the number of neurons in the hidden layer, *nFeats* is the number of features and *nTrees* is the number of trees

The performance evaluation is assessed in terms of detection in the *clean* class, so a true positive (*TP*) is referred to as the correct classification of a clean segment, and a false negative (*FN*) to its misclassification. Regarding the *noisy* class, a true negative (*TN*) stands for the correct identification of a noisy segment, whereas a false positive (*FP*) occurs when it is classified as clean. Five standard metrics are employed to estimate the performance. The probability of correctly classifying any signal block is measured by means of the accuracy:

6 $$ Acc = \frac{TP+TN}{TP+FN+TN+FP} $$

Recall is used to assess the probability of correct classifications in the *clean* class

7 $$ Re=\frac{TP}{TP+FN} $$

The probability of a signal segment classified as clean being correct is evaluated through the precision:

8 $$ Pr=\frac{TP}{TP+FP} $$

As a balance measure between precision and recall, F1 score is assessed:

9 $$ F1 = 2\cdot\frac{Re \cdot Pr}{Re+Pr} $$

At last, we also employ the Matthew Correlation Coefficient (MCC):

10 $$ MCC= \frac{TP\cdot TN-FP\cdot FN}{\sqrt{(TP+FP)(TP+FN)(TN+FP)(TN+FN)}} $$

The MCC eases evaluating and comparing the performance of binary classifiers when the classes are unbalanced [39]. When comparing classifiers, the MCC is the only score that improves only if the prediction improves simultaneously for the positive and the negative class instances. In this work, the normalized version of the MCC is used $nMCC = \frac {MCC+1}{2}$, such that *nMCC* ∈ [0,1].

## Results

### Performance of the models

**Fig. 5:**
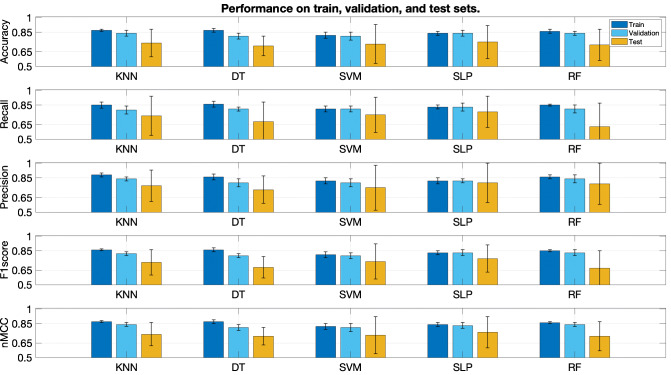
Classification performance with different ML methods. Results are shown in terms of mean and standard deviation. Each graph corresponds to one metric, namely (from top to bottom), accuracy, recall, precision F1, and nMCC. Each bunch of three bars corresponds, from left to right, to the train, validation, and test sets

Each graph of Fig. 5 shows the performance for each metric. Each bar triplet corresponds to the results on the three sets (from left to right: train, validation, and test, respectively). Each bar stands for the mean value, and the standard deviation is represented as whiskers around the mean. In general, the performance is similar, as can be seen in the very close values attained by *nMCC* (bottom graph). The classification performance in the train and validation sets always outperforms that of the test set in all the cases, which confirms the overestimated performance when the same patients are used for testing. There are two models where differences between validation and test are smaller, namely, SLP and SVM (third and fourth columns of each graph).

Regarding the variability, standard deviation is significantly greater in the test set than in the other sets for all the cases. Reasons are twofold. First, the limited extent of the database, which fails to learn all the inter-subject variability, resulting in poorer performance when the model is applied to unknown data. Second, the particular characteristics of one single individual in the test set may not be necessarily well represented in the training set, inducing higher variability when changing the patient.

The best method is chosen according to the next considerations: small performance differences among the three sets, indicative of less overfitting to the train set; small variability in the test set, indicative of robustness, since lower standard deviation suggests similar behavior for the different patients in the test set; and good trade-off between recall an precision. The smallest deviations in the test set are obtained by *K* NN, DT, and SLP (first, second, and fourth columns), but the best deal among all the previous considerations is given by the SLP, as it yields better recall and precision in the test set. The detailed performance of the SLP is shown in Table 4 (rows 2 to 3).

**Table 4:** Classification performance using the SLP

	*Acc*	*Re*	*Pr*	*F*1	*nMCC*
Train	0.84 ± 0.02	0.83 ± 0.02	0.82 ± 0.03	0.83 ± 0.02	0.84 ± 0.02
Validation	0.84 ± 0.03	0.83 ± 0.04	0.82 ± 0.02	0.83 ± 0.03	0.83 ± 0.03
Test	0.75 ± 0.17	0.78 ± 0.16	0.80 ± 0.20	0.77 ± 0.14	0.76 ± 0.16
Test with 9 features	0.75 ± 0.16	0.79 ± 0.15	0.77 ± 0.18	0.77 ± 0.12	0.75 ± 0.14
Test with 8 features	0.73 ± 0.17	0.73 ± 0.21	0.78 ± 0.19	0.73 ± 0.14	0.74 ± 0.15

Table 5 shows the SLP performance for each patient in the test set, reporting the confussion matrix (letters *c* and *n* refer to *clean* and *noisy* classes, respectively). The minimum and maximum *F*1-score values are attained for the models tested on patient 6 (0.54) and patient 4 (0.95), respectively. The low performance of patient 6 indicates that the characteristics are not learned from the other five.

**Table 5:** Detailed classification results for each SLP

Test		Real		*Acc*	*Re*	*Pr*	*F*1	*nMCC*
		*c*	*n*					
	Predicted							
**2**	*c*	174	52	0.81	0.72	0.77	0.75	0.79
	*n*	67	320					
**4**	*c*	124	10	0.97	0.97	0.93	0.95	0.96
	*n*	4	371					
**6**	*c*	180	243	0.45	0.73	0.43	0.54	0.48
	*n*	66	76					
**7**	*c*	211	79	0.75	0.99	0.73	0.84	0.72
	*n*	3	36					
**9**	*c*	92	4	0.76	0.59	0.96	0.73	0.80
	*n*	64	128					
**10**	*c*	223	12	0.76	0.67	0.95	0.79	0.79
	*n*	109	170					

Letters *c* and *n* refer to *clean* and *noisy* classes, respectively

### Feature selection

**Fig. 6:**
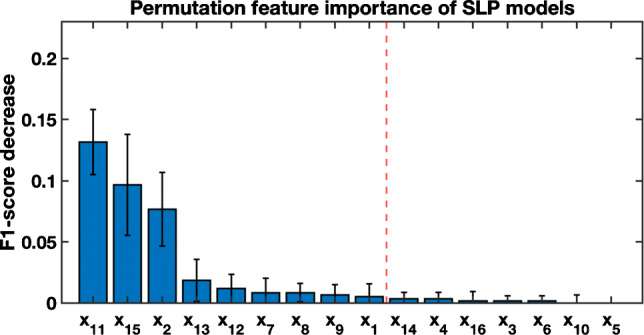
Permutation feature importance. The broken red line separates the most important individual features for the SLP models

The impact of each individual feature is evaluated by applying *permutation feature importance* [33, 40], which consists of analyzing the decrease in performance when a feature *x*_*j*_ is randomly shuffled. Therefore, the decrease in performance becomes an indication of how dependent the model is on that feature because the relationship between that feature and the categories is broken. Figure 6 shows the results of sorted features from highest to the lowest importance for SLP model. The first three are the most significant, though the decrease in performance is not significant. Table 4 shows the results of the SLP with 9 and 8 features (rows 4 and 5). As can be seen, the performance with 9 features is quite similar to the performance with all features. Not only can the complexity of the model be reduced by using fewer features without losing classification capability, but also it is possible to eliminate redundant or useless information for the model.

### Performance on ECG inline

**Fig. 7:**
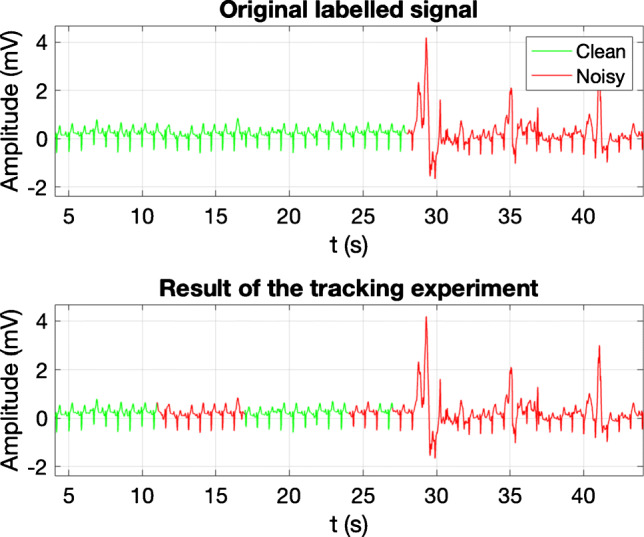
Example of noise tracking. The top graph shows an original labelled excerpt that corresponds to 50 s taken from an LTM signal. It presents a transition from clean to noisy class, at approximately *t* = 28 s. The bottom graph shows the classification result obtained by the ML system

An example of actual working conditions is carried out with the SLP on the whole original EER database introduced in Section 2.1. Signals are processed with a 5-s length sliding window and 4-s overlap. The 250 signals were split in two bunches: Bunch 1 (B1) gathers the 6 patients used to train the system as previously described; and bunch 2 (B2) with the excluded patients. Although it was expected a better performance in B1, results are similar (see Table 6), because most of the parts of the ECGs from B1 were not selected for the training dataset. A visual example is shown in Fig. 7, which depicts an excerpt of 50 s taken from one ECG of B2 with a transition from *clean* to *noisy* classes at, approximately, *t* = 28 s. The result of the tracking experiment, in the bottom graph, shows one segment misclassified as *noisy* in the first 24 s of the excerpt. The result around *t* = 28 s reveals that a running and continuous tracking application may have some difficulty in finding the exact point where classes change.

**Table 6:** Performance results of the SLP operating in running conditions on actual signals with class transitions

	*Re*		*Pr*		*nMCC*	
	lead_1_	lead_2_	lead_1_	lead_2_	lead_1_	lead_2_
**B1 (2h, 50’ 41”)**	0.85	0.76	0.85	0.69	0.81	0.77
**B2 (3h, 36’ 43”)**	0.88	0.92	0.65	0.74	0.77	0.83

B1 stands for the set of ECGs from the 6 training patients and B2 for the bunch with the remaining 4 patients with class imbalance

## Discussion

There are several works which address the problem of noise in ECG using ML techniques to assess different scales of severity such as the one proposed in this project. Most of the approaches based on realistic ECG recordings of short duration [24–26] extract signal quality indexes to feed different ML classifiers. Others focus on exploiting the structure of the cross-covariance matrix [22] or the autocorrelation [21] among short ECG segments to compute the features. The addition of synthetic noise at different scales of severity to the ECG blocks is the technique most commonly used to assess the performance of the algorithms. These previous works make a characterization of the noise in ECG in terms of quantitative severity, except [26], which uses a 5-level scale of clinical severity, but it also performs the validation on a database of ECG segments with additive noise. Alternately, an approach with actual LTM recordings is addressed in [20], though the quality scale is not defined. So, to the best of our knowledge, none of the previous works have developed a gold standard relying on qualitative severity. Conversely, in this paper we propose the characterization of noise according to a clinical noise severity [10], using for this purpose a manually annotated database. The main difference of this work with regard to the previous ones is that we deal with LTM data acquired in real conditions and continuously labeled from a clinical perspective. Besides, the data is labeled according to clinical noise criteria ensuring that the ECG waveform fulfills the conditions to contain interpretable information for diagnostic.

The size of the database is a limitation of this work because it only covers a time duration of approximately 6.5 h of only 10 patients. This restriction is further affected by the segmentation process carried out to design a suitable dataset to validate learning systems along with the necessity of making class balancing. This process leads to a reduction of both the database size and the number of patients. However, the major value of the database lies in its annotations, a continuous running labelling of all the ECG records set to be a gold standard to assess qualitative noise severity. So, with this powerful tool at hand, and despite the available reduced information, the application of a solid methodology supports the hypothesis that clinical noise classes can be distinguished using ML techniques. The soundness of the procedure relies on the following considerations: selection of independent ECG segments to design the dataset, patient withdrawal in favor of data balance, patient separation to test, and design of different optimized models to be evaluated on different test sets. Despite this solid experimental framework setting, the limitation of the database constrains the performance to a moderate-to-high values of 0.78 and 0.80 in recall and precision, respectively, though this is also a proof that there is room for the improvement.

Increasing the database size may be a question to be further addressed, as it would surely contribute to outperform the reported results and also to employ models based on deep learning. Unfortunately, the design of synthetic data is significantly constrained, since the impact of clinical noise is not assessed by quantitative means. One possibility is to assemble new annotated data of running and continuous labelling, but this process is extremely cumbersome and time consuming, so it does not seem to be the most feasible way. Furthermore, the segmentation applied to the database, as reported in Section 2, wastes valuable information, such as annotated segments of less than the selected *t*_*b*_ time length or data imbalance. Rather than dedicating loads of human hours in manually labelling ECG, we believe that an incremental method combining labelling with the use of a trained model would be more beneficial. Thus, we consider that techniques such as Active Learning [41, 42], which allows short-term monitoring, increasing the amount of labelled data and calibration of the improvement at the same time, should be a better choice.

**Fig. 8:**
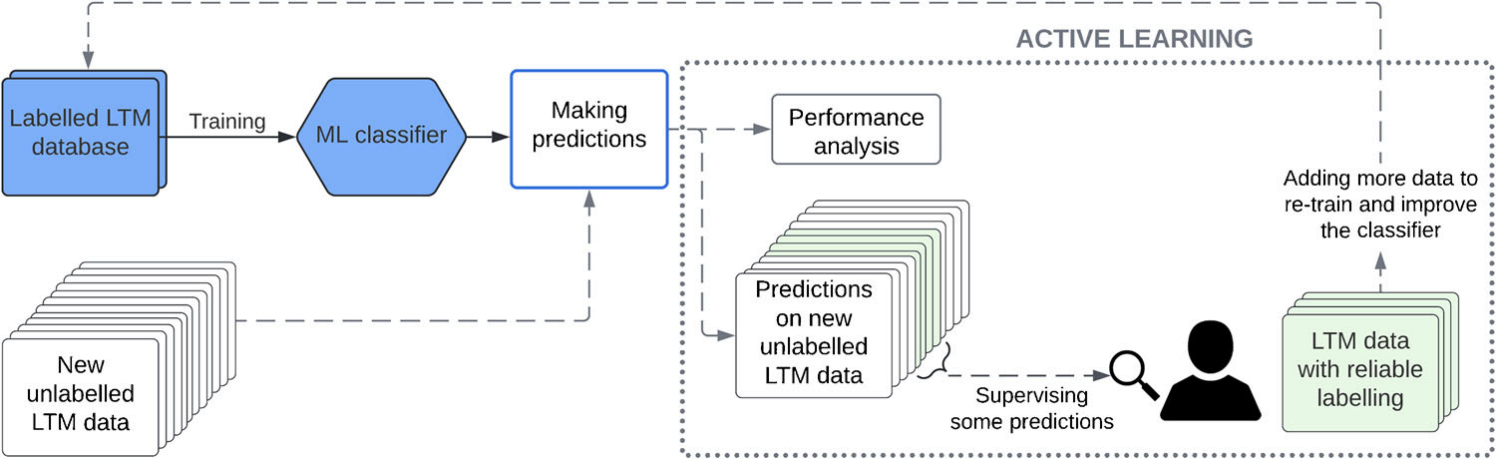
Active learning scheme: iterative method for database growth and performance improvement

A possible approach to address an Active Learning methodology is shown in the diagram of Fig. 8, where the classifier trained in the present work (blue boxes) is the seed to this technique. From this point, the main idea is to make predictions on new unlabelled LTM data with the classifier. Once the predictions have been made, a user reviews only a portion of these predictions (green), e.g., those where the classifier fails the most and, therefore, their correction and inclusion in classifier retraining will help to further improve its performance. Hence, the classifier learns, not only from the initial labelled LTM database, but also from new data in each iteration. This feedback will allow the classifier to improve its performance and also its predictions on large amounts of unlabelled data. As it can be seen, it is an iterative process where the evolution of the classifier performance is also analyzed. Thus, after several iterations, it would be possible to obtain a fairly reliable labeling on the initially unlabelled LTM database. This approach could solve the current database size limitation and also would lead to the use of deep learning-based models, such as convolutional neural networks, for which a large amount of labelled data is needed.

In the experimental setting, we have decided to rotate patients in the test set to check the consistency. So, for a particular learning technique, we expected to see some uniformity of the models optimized with different hyperparameter when operating on new data. The results exhibited in Fig. 5 indicates high variability of the estimated performance (test set). One reason for that may be due to insufficient number of examples to train. Another one can be given by the inter-patient variability, which is clearly patent in Table 5, where it can be observed that the classification results vary significantly depending on which patients are included in the training and test set. All these results indicate that the approach tackled in the work to characterize clinical noise is promising, but that the performance improvement is subject to the arrangement of a larger database with more patients. Another important issue to be highlighted is preventing patient-overfitting. Figure 5 exhibits a performance drop between the validation and the test sets. The reason is due, in part, to the fact that the validation and the training sets share patients, i.e., although they contain different instances, they come from shared patients. So, the validation set cannot be considered as new data to the classifiers.

Initially, the original split of clinical noise was defined in a five classes scale. However, the main objective in this field is to discern which parts of an ECG record can be used to obtain a diagnosis. Given that the original taxonomy relies on what clinicians interpret as noise in an ECG, the different categories can be gathered into two groups to achieve this goal, as carried out in this work. Thus, subsequent analysis stages can use the output of the noise characterization system for different several purposes, e.g., either automatically process or manually supervise ECG regions with diagnostic potential. According to this, we have proposed in this work to reduce the multi-class problem to a binary one by including noise levels T2 and T3 into the non-valid or *noisy* category. The latter because it represents a signal with no recognizable ECG patterns at all, and the former, because it hardly provides the rhythm identification. We are aware that this distribution is not unique, and that many other possibilities are feasible. For example, class T2, chosen as noisy in this work, can very well be used for Heart Rate Variability studies, though we understand that the distinction between T2 and T3 is even a simpler problem than the one conceived in this work.

With the increasing number of e-health devices, signaling whether the collected data has value, which is indeed linked to the quality of the data, is of utmost importance due to the possible overwhelming quantity of data. Even for automated analysis and diagnosis, a first step would be restricting the analysis to interpretable segments to save time, energy, and other resources. This entails a binary classification. Our contribution is a first approach targeting this (engineering) problem evaluating its possibility, and discussing the necessary steps in this direction.

## Conclusions

The results of this work show that the use of learning-based systems to solve a problem of clinical noise classification in electrocardiography on a binary dimension scale is possible. The procedure developed to prove the feasibility of ML classifiers to assess clinical quality in the ECG relies on a robust methodology which takes into account balance of classes and inter-patient variability to conduct experiments with five different ML classifiers. The gold standard is a LTM database entirely annotated according to a clinical noise taxonomy. All the models fitted into these data have proven nice performance in classifying ECG signal frames with diagnostic capability. Regarding the limitation of the current work, the improvement is subject to further explorations on advanced and semi-supervised techniques to gather additional and reliable labelled data. Methods such as active learning may play an important role to outperform the detection ability of learning systems and to increase the amount of labelled data within the framework of these long–duration signals.
